# Incubation Period and Serial Interval of Mpox in 2022 Global Outbreak Compared with Historical Estimates

**DOI:** 10.3201/eid3006.231095

**Published:** 2024-06

**Authors:** Luis Ponce, Natalie M. Linton, Wu Han Toh, Hao-Yuan Cheng, Robin N. Thompson, Andrei R. Akhmetzhanov, Jonathan Dushoff

**Affiliations:** National Taiwan University, Taipei, Taiwan (L. Ponce, A.R. Akhmetzhanov);; California Department of Public Health, Richmond, California, USA (N.M. Linton);; Johns Hopkins University, Baltimore, Maryland, USA (W.H. Toh);; Taiwan Centers for Disease Control, Taipei (H. Cheng);; University of Oxford, Oxford, UK (R.N. Thompson);; McMaster University, Hamilton, Ontario, Canada (J. Dushoff)

**Keywords:** mpox, monkeypox virus, viruses, global outbreak, incubation period, serial interval, rash

## Abstract

Understanding changes in the transmission dynamics of mpox requires comparing recent estimates of key epidemiologic parameters with historical data. We derived historical estimates for the incubation period and serial interval for mpox and contrasted them with pooled estimates from the 2022 outbreak. Our findings show the pooled mean infection-to-onset incubation period was 8.1 days for the 2022 outbreak and 8.2 days historically, indicating the incubation periods remained relatively consistent over time, despite a shift in the major mode of transmission. However, we estimated the onset-to-onset serial interval at 8.7 days using 2022 data, compared with 14.2 days using historical data. Although the reason for this shortening of the serial interval is unclear, it may be because of increased public health interventions or a shift in the mode of transmission. Recognizing such temporal shifts is essential for informed response strategies, and public health measures remain crucial for controlling mpox and similar future outbreaks.

Mpox, caused by monkeypox virus (MPXV), is a viral illness characterized by rash, influenza-like symptoms, and fever. A global outbreak of mpox attracted increased public attention in 2022 and became recognized as a public health event of international concern (PHEIC). Historical estimates of the case-fatality ratio (CFR) associated with mpox infection vary by clade; clade I exhibits a CFR of »10%, whereas clade II the CFR is <1% (*1*). Although mpox historically experienced limited transmission (*2*,*3*), the 2022 outbreak, originating in nonendemic countries in Europe and North America, resulted in »90,000 cases by mid-April 2023 and demonstrated enhanced transmissibility (*4*). The outbreak was driven primarily by sexually associated transmission, which altered the clinical manifestations and epidemiology of the infections when compared with historical reports (*5*). Clade II was dominant; its case-fatality ratio was »0.1% (*6*). Although certain epidemiologic parameters, such as the incubation period and serial interval, have been estimated using case records from 2022 (*7**–**13*), comprehensive analysis of historical estimates and assessment of their relationship to the recent outbreak is limited (*14*).

After MPXV was identified in imported monkeys in Denmark in 1958, reported mpox infections were frequently associated with contact with monkeys (*15*–*17*). However, subsequent findings revealed that primates are not the only reservoir hosts (*18*). Before the eradication of smallpox in 1980, mpox was rarely observed in humans, in part because mpox is unlikely to have been widespread but also because of cross-immunity between the 2 viruses. The mpox outbreaks in the 1970s–1990s were relatively small in scale, typically involving <5 cases, and predominantly affected children because most adults possessed some level of immunity from smallpox infection or vaccination (*18*). However, as herd immunity waned, outbreaks in the 2000s caused dozens of cases (*19*,*20*); mpox became endemic in some regions of Africa, and Nigeria reporting the largest outbreaks (*21*,*22*). 

The first major outbreak reported beyond the borders of Africa occurred in the United States in 2003; there were 81 confirmed cases linked to imported wild animals (*23*). The global outbreak in 2022 caught many by surprise as mpox spread rapidly in countries across Western Europe and North America in which it was not endemic, before expanding worldwide. The World Health Organization (WHO) declared the 2022 mpox outbreak a PHEIC on July 23, 2022 (*24*). By early 2023, case numbers had begun to decline, likely because there were fewer highly connected susceptible persons within sexual networks (*25*). In addition to the depletion of susceptible persons, general behavioral changes in high-risk populations resulting from increased awareness of risk and vaccination of at-risk persons played an important role in the decline in mpox cases (*26*). Modeling of infections caused by sexual interactions among men who have sex with men (MSM) has shown that having fewer 1-time partnerships can significantly reduce mpox transmission (*27*). Furthermore, members of higher-risk populations proactively altered their behaviors in response to the outbreak; many were vaccinated. In August 2022, a survey of MSM in the United States revealed that »50% had reduced their use of dating apps, number of sexual partners, and number of 1-time partnerships (*28*).

The clinical manifestation of mpox has historically been similar to that of smallpox or chickenpox, characterized by fever, rash, and lymphadenopathy (*1*). Its distinctive rash initiates as macules and progresses through papules, vesicles, pustules, and crusts before resolving. Lymphadenopathy, reported in 85% of mpox cases (*29*), distinguishes mpox from smallpox and chickenpox. Some mpox patients also exhibit respiratory symptoms such as sore throat, nasal congestion, or cough.

Since 2022, some changes in the clinical manifestations of mpox have been observed (*5*), including a tendency for skin lesions to localize to specific body regions associated with sexual transmission, such as the genital, anorectal, or oral areas. Rectal symptoms such as purulent or bloody stools, rectal pain, or bleeding were frequently reported (*30*). Some patients exhibited only a few cutaneous formations near affected areas, whereas others experienced disseminated body rashes complicating their infection. Although the localized rash may appear almost concurrently with other initial symptoms, the disseminated rash usually appeared several days after symptom onset.

Some estimates of the incubation period and serial interval for the global mpox outbreak in 2022 have been affected by right-truncation bias. This bias arises when only persons who have experienced the event (e.g., symptom onset or rash appearance) and were confirmed by testing at the time of data collection are included in the sample. By accounting for right truncation, we can estimate the length of the incubation period and serial interval more accurately and include cases with symptoms who have not yet been reported. Ignoring right truncation leads to underestimation of such epidemiologic parameters, because cases with longer incubation periods or serial intervals are overlooked in the analysis. Earlier studies reported short mean incubation period estimates of 9.0 days (*7*) and 7.6 days (95% credible interval [CrI] 6.5–9.9 days) (*10*), extended to 9.5 days (95% CrI 7.4–12.3 days) when accounting for right truncation (*10*).

Estimation of the incubation period of mpox presents several difficulties. One challenge arises from the absence of definitive information on times of exposure. The exposure time window for much recorded data was often >1 day, complicating estimation. Excluding records with longer windows may yield biased estimates, as we saw in lower estimates from the exclusion-based approach (*31*) compared with other studies (*32**,**33*). Furthermore, some studies calculated the incubation period from the last known time of contact (*5*) instead of considering the entire exposure period, which also led to underestimation of the true incubation period.

Estimating generation time or serial intervals (time intervals from an event in an infector to the same event in an infectee) for the historical period before 2022 presents even greater uncertainty. As of April 2024, we are aware of no published formal estimates of such intervals from historical data, although estimates for the global 2022 outbreak exist; 34 transmission (infector‒infectee) pairs studied in the Netherlands yielded a mean onset-to-onset serial interval estimate of 10.1 days (95% CrI 6.6–14.7 days) (*9*), and another estimate of 9.5 days (95% CrI 7.4–12.3 days) was based on 79 transmission pairs notified in the United Kingdom (*10*). In contrast, limited information on transmission pairs is available for the pre-2022 period; researchers observed onset-to-onset intervals of 8‒11 days (*34*,*35*). We analyzed additional published data from before 2022 for rash-to-rash (*2*) and onset-to-onset serial intervals (*19*,*20*,*35*). The aim of our research is to provide historical estimates of the epidemiologic parameters associated with mpox by aggregating available historical data and to compare those estimates with pooled estimates for the global 2022 outbreak. In our analysis, we corrected previous estimates as appropriate to account for right truncation, enabling systematic comparison of incubation periods and serial intervals across the 2 time periods. Our work did not require the approval of an ethics committee because it was based on a literature search and the analysis of publicly available data.

Despite successful containment of mpox in 2022–2023, monkeypox virus has continued to spread via human-to-human transmission worldwide. Investment in mpox surveillance and prevention methods, including vaccination, are critical to prevent the virus from causing future outbreaks and reaching PHEIC status again. As emphasized by WHO (*24*), it is necessary to remain vigilant and implement preventive measures to stop mpox from becoming endemic worldwide. Improving available knowledge of the epidemiologic parameters characterizing transmission, such as the incubation period and serial interval, represents a fundamental aspect of this global effort.

## Methods

### Epidemiologic Data

**Figure 1:**
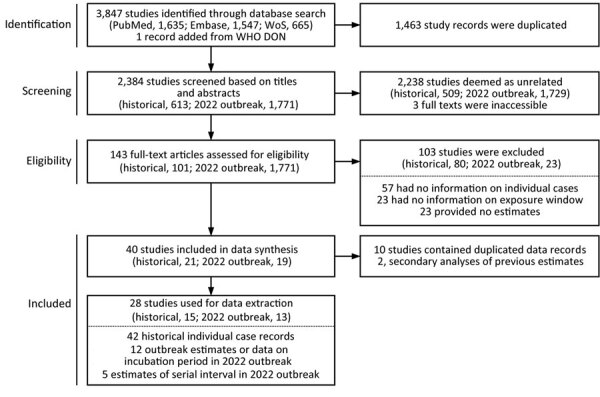
Flow diagram describing identification of historical case records from before the 2022 mpox outbreak eligible for estimation of the incubation period and studies reporting estimates of the incubation period and serial interval during the 2022 outbreak. WoS, Web of Science.

We conducted a comprehensive literature search without language restriction using the electronic databases PubMed, Embase, and Web of Science through January 4, 2024. We searched for the terms monkeypox, mpox, or mpx, and >1 occurrence of the terms incubation, serial, symptoms, onset, or rash. We extracted individual case records of infections from the studies published before 2022 and extracted estimates of the incubation period and serial interval from the studies published after 2022. The search yielded a total of 2,384 references after deduplication (Figure 1). 

We deemed a total of 101 references published before 2022 relevant for collection of historical data after manual examination. We found specific information on dates of exposure and symptom onset in 21 references. We excluded 6 studies containing duplicate data. Ultimately, we selected 15 studies with a total of 42 case records. Of those, 16 records were associated with clade I MPXV, and all contained information on rash and symptom onset date; 26 records were associated with clade II, and 12 had information on rash and symptom onset date.

Among manuscripts published after 2022, we deemed 42 relevant after manual inspection. We retrieved 12 estimates of the incubation period and 5 estimates of the serial interval from studies providing data from Colombia (*36*), Italy (*8*), the Netherlands (*7*,*9*), Nigeria (*37*), Spain (*5*,*38*), the United Kingdom (*10*,*39*), and the United States (*11*), as well as studies providing data from multiple countries (*12*,*13*,*40*). Three of those publications included estimates that adjusted for right truncation of the data. To account for right truncation in estimates from the other studies, we extracted individual case data from published materials or obtained the data from the authors. We also compared the extracted list of publications with the literature search conducted by WHO as of December 29, 2022 (*41*). Some references listed by WHO were not identified in our search because they were posted on a preprint server and not peer reviewed by the time of our assessment. (Appendix Table 1). 

### Statistical Analysis

We estimated the incubation period and serial interval distributions using a Bayesian model with Markov chain Monte Carlo implemented in Stan version 2.34.0 (https://mc-stan.org). We used the generalized gamma distribution to determine the incubation period and serial interval because it encompasses 3 commonly used distributions (gamma, Weibull, and log-normal) (*42*). We considered alternative formulations using standalone gamma, Weibull, or log-normal distributions or their mixture and saw no clear differences in the results (Appendix Figure 2).

For identified studies from the 2022 outbreak that did not account for right truncation (*7*,*8*,*11*), we extracted case data. In 2 of those studies (*7*,*8*), the authors provided the truncation date (the final day that case data were available)—day 38 (*7*) and 68 (*8*). With those dates, we could re-estimate the incubation period and serial interval accounting for right truncation. However, in 2 studies (*11*,*39*), no information about truncation date was available, so we were unable to conduct a re-analysis to account for right truncation. The authors of those studies stated that they observed no significant difference between nontruncated and right-truncated likelihoods. We obtained a pooled estimate of the mean incubation period from the meta-analysis using a random-effects model (*43*).

To estimate historical serial intervals, we used data from published studies (*2*,*19*,*20*,*35*). We extracted rash-to-rash time intervals from the dataset provided by Jezěk et al. (*2*) and onset-to-onset intervals (based on generalized symptoms) from other sources (*19*,*20*,*35*); the result was available data from 28 transmission pairs. Consistent with the discussion in Jezek et al., we omitted rash-to-rash intervals of <8 days, which likely resulted from co-primary infections. 

To ensure the robustness of our estimates, we conducted a sensitivity analysis (Appendix). First, we considered different cutoff values (2, 4, 6, or 10 days), below which the rash-to-rash intervals were omitted. Second, we fitted the observed distribution to a composition of 2 distributions to allow for the possibility that cases with serial intervals longer than the cutoff value could still be co-primary infections. The first component was modeled either by an exponential distribution or by a scaled standard normal distribution, normal(0, σ), in line with previous studies. The second component was the rash-to-rash serial interval of interest, which was modeled by the generalized gamma distribution.

## Results

**Figure 2:**
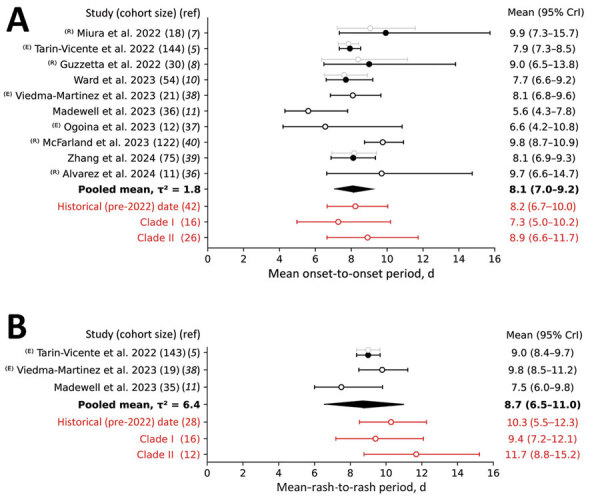
Forest plot of the mean infection-to-onset (A) and infection-to-rash (B) incubation periods for studies conducted during the 2022–2023 global mpox outbreak and analyses of the historical case records. Open circles indicate analyses performed without adjusting for right truncation (ICC); solid circles indicate analysis when an adjustment was made (ICRTC). Whiskers indicate 95% CrIs. Studies are denoted by the leading author and year of publication and ordered by their date of publication; the numbers in parentheses indicate the number of case records used for estimation. ^(E)^ indicates that we evaluated the estimates using the data provided in our study; ^(R)^ indicates that we re-evaluated estimates for consistency of the methods used. Gray indicates estimates not used for deriving the pooled mean, which is in bold text. Red indicates estimates for historical (pre‒2022 outbreak) data, indicating that they were not used for deriving the pooled mean. CrI, credible interval; ICC, interval censoring corrected model; ICRTC, interval censoring and right truncation corrected model; ^2^ = -squared statistics indicating the between-study variance measured in days^2^; ref, reference.

We report estimates of the mean and SD of the incubation period for mpox based on recent literature (Figure 2, panel A; Appendix Table 2). We obtained those estimates in various ways. For 2 previous studies, we re-derived the estimates in the original articles to account for right truncation (*7*,*8*). We obtained other estimates by either fitting our model to data from the original publications (*5*,*36*,*38*) or reporting the findings from the original studies directly (*10*,*11*). The pooled mean incubation period was estimated to be 8.1 days (95% CrI 7.0–9.2 days); here, we reported all estimates as the posterior median and 95% CrI. The mean between-study variance was 1.8 days^2^. Analysis of historical data (before the 2022 outbreak) suggested a mean incubation period of 8.2 days (95% CrI 6.7–10.0 days). Considering only cases associated with clade I resulted in a slightly lower mean of 7.3 days (95% CrI 5.0–10.2 days), whereas clade II infections were characterized by longer mean of 8.9 days (95% CrI 6.6–11.7 days). The 95th percentile of the incubation period distribution, commonly used to determine the quarantine period, was 16–20 days across all studies of the global 2022 outbreak and was 17 days for the historical data.

We also assessed the infection-to-rash incubation period, which tracks the time from infection to the manifestation of a cutaneous rash (Figure 2, panel B; Appendix Table 3). We estimated the pooled mean as 8.7 days (95% CrI 6.5–11.0 days), whereas the between-study variance was 6.4 days^2^. Historical data gave a larger estimate of 10.3 days (95% CrI 8.5–12.3 days). We reviewed 3 studies for the 2022 outbreak; Madewell et al. (*11*) estimated a mean incubation period substantially lower than 2 other studies that looked at infection-to-rash time intervals (*5*,*38*), which resulted in a larger discrepancy between the pooled mean and historical estimate compared with the infection-to-onset incubation period estimates. Rash emergence was delayed by a mean of 0.6 days, compared with the infection-to-onset incubation period. Analyzing the data from Viedma-Martinez et al. (*38*), we first calculated the time from infection to the appearance of any cutaneous formations to have a mean value of 9.8 days (95% CrI 8.5–11.2 days). We then calculated the time from infection to the appearance of a disseminated rash, excluding the rash around or at the site of infection. We estimated a mean time period of 11.5 days (95% CrI 10.0–12.8 days). The difference between initial onset of symptoms and rash onset was 1.7 days (95% CrI 0.2–3.7 days) when considering a localized rash and 3.4 days (95% CrI 1.4–5.3 days) when considering a disseminated rash.

**Figure 3:**
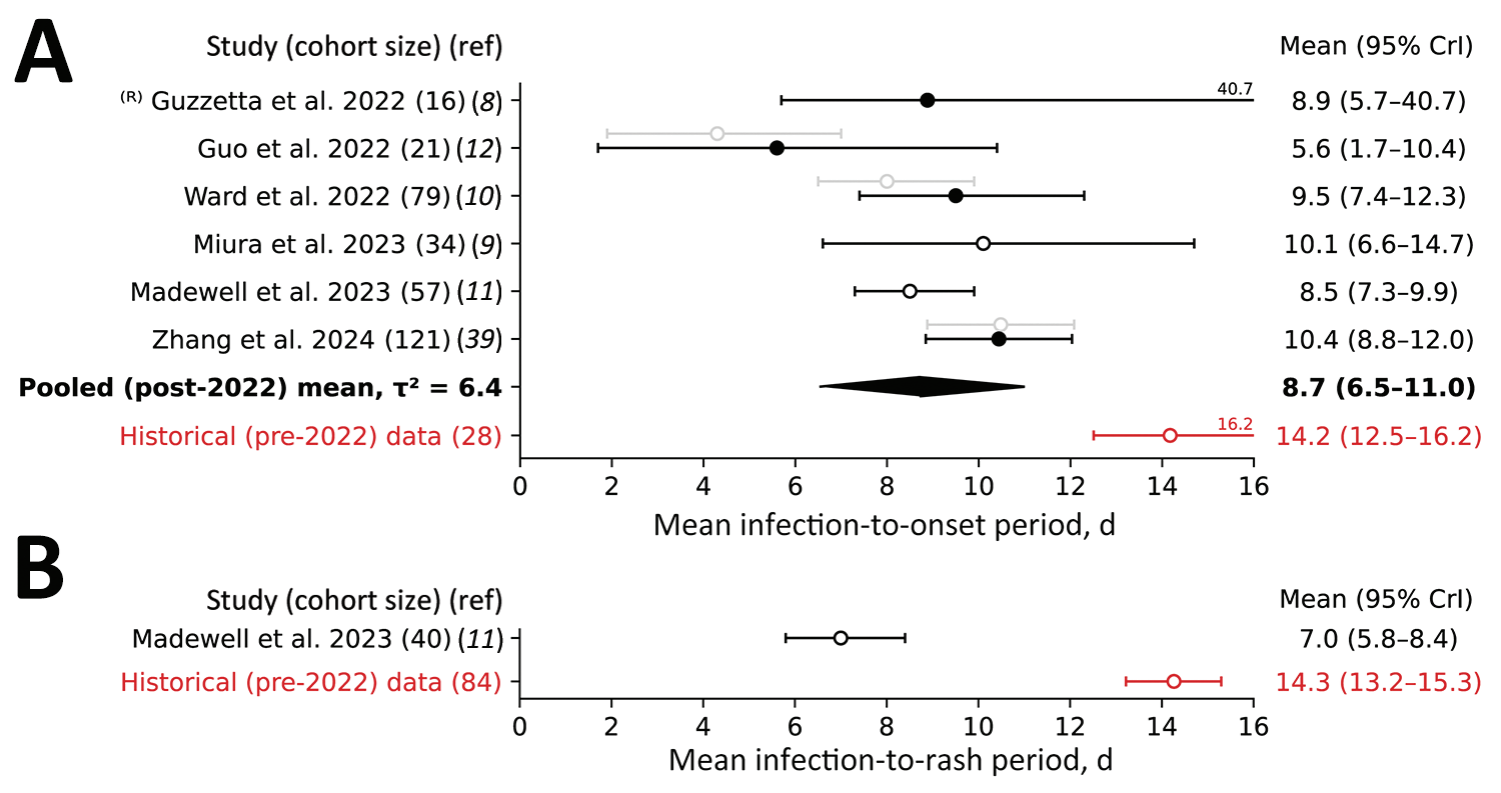
Forest plot of the estimated mean serial interval based on the date of symptom onset (A) and the date of rash onset (B) for studies conducted during the 2022–2023 global mpox outbreak and analyses of the historical case records. Open circles indicate analyses performed without adjusting for right truncation (ICC); solid circles indicate analyses when an adjustment was made (ICRTC). Whiskers indicate 95% CrI. Studies are denoted by the leading author and year of publication and ordered by their date of publication; the numbers in parentheses indicate the number of case records used for estimation. ^R)^ indicates that we re-evaluated estimates for consistency of the methods used. Gray indicates estimates not used for deriving the pooled mean, which is in bold text. Red indicates estimates for historical (pre‒2022 outbreak) data, indicating that they were not used for deriving the pooled mean. CrI,  credible interval; ICC, interval censoring corrected model; ICRTC, interval censoring and right truncation corrected model; ref, reference; ^2^, -squared statistics indicating the between-study variance measured in days^2^.

As for incubation period estimates, serial interval estimates varied substantially across studies. We estimated the pooled mean for onset-to-onset serial intervals as 8.7 days (95% CrI 6.5–11.0 days) and between-study variance as 6.4 days^2^. We estimated the historical mean onset-to-onset serial interval at a much longer 14.2 days (95% CrI 12.5–16.2 days) (Figure 3, panel A; Appendix Table 4, Figure 1, panel A). Madewell et al. (*11*) reported rash-to-rash serial intervals for the 2022 outbreak; they reported a mean of 7.0 days (95% CrI 5.8–8.4 days). That value is much shorter than the historical estimate of the mean rash-to-rash serial interval, which was 14.3 days (95% CrI 13.2–15.3 days) (Figure 3, panel B; Appendix Table 5, Figure 1, panel B). Although Madewell et al. suggested that serial intervals might be shorter than incubation periods for the 2022 outbreak, we found that serial intervals were substantially longer for historical data. Specifically, our analyses suggested that onset-to-onset serial intervals were on average 6.0 days longer than for infection-to-onset incubation periods, and rash-to-rash serial intervals were on average 4.0 days longer than for infection-to-rash incubation periods.

## Discussion

In this study, we undertook a systematic literature search and meta-analysis to provide estimates of the incubation period and serial interval of mpox. We compared estimates from the 2022 outbreak with pre-2022 estimates. We found a strong similarity in estimates of infection-to-onset and infection-to-rash incubation periods between studies for the 2022 outbreak and historical case records. However, the serial interval estimates based on historical data were longer than the incubation period estimates based on historical data, which suggests a lower risk of presymptomatic transmission during the pre-2022 period. The shorter serial interval observed in the 2022 outbreak might also be partially attributable to nonpharmaceutical interventions such as contact tracing, active case finding, and behavioral changes, as noted during the COVID-19 pandemic (*44*). A shift toward a sexually associated mode of transmission as the dominant route may also have influenced the serial interval, perhaps by increasing transmission efficiency. All of those theories merit further investigation.

The estimated incubation period in this study remains similar to historical estimates (*45*), suggesting that the recommended quarantine period of 21 days after contact with a potential infector is still appropriate. However, the possible increase in presymptomatic transmission, as suggested by a shortened serial interval, presents challenges for successful containment of future outbreaks (*9*,*11*). Moreover, underascertainment of cases further reduces the chances of efficient case finding and contact tracing. Vaccination is regarded as the most reliable measure to prevent future waves of infections, but vaccine availability and uptake have been limited. Some countries that observed a spike in cases in 2022 saw their outbreaks fade in 2023, but other countries in the Western Pacific region, such as Japan, South Korea, and Taiwan, observed a rise in cases at the beginning of 2023 (*46*).

The 2022 global mpox outbreak shares some similarities with a previous outbreak in Taiwan involving a sexually transmitted pathogen that also affected a vulnerable group. In 2015–2016, hepatitis A virus (HAV) infections spread progressively among the MSM population. The Taiwan Centers for Disease Control (CDC) reported an increase in HAV cases in 2015. A free HAV vaccination campaign was initiated in October 2016, several months after the peak of disease incidence, targeting at-risk populations. Because it was difficult to quantify the direct impact of vaccination after the peak on the course of the outbreak, many attributed the decline in cases to the promotion of both HAV screening and vaccination by physicians earlier in the outbreak (*47*). In the 2022 global mpox outbreak, there has been much debate about the key factors behind the decline in incidence observed in all hard-hit countries in mid-to-late 2022. Some suggested depletion of susceptible persons within sexual networks of MSM was the key factor (*25*); others argued that a synergetic effect of behavioral change and vaccination was crucial (*48*). Going forward, proactive vaccination campaigns are advised to reduce transmission; such a campaign was implemented in Taiwan at the beginning of 2023 after reports of locally acquired mpox infections.

The first limitation of this study is that we derived the pooled estimates of the mean incubation period and serial interval from various sources, each with their own potential biases and limitations. For example, the study by Ward et al. (*10*) did not consider the possibility of co-primary cases; however, it used personally identifiable information to establish linked pairs. Second, the aggregated historical data could also be prone to selection and recall biases; many studies were conducted retrospectively, and mild cases may have been missed. Third, most cases in the historical datasets involved children and teenagers, whereas in the 2022 outbreak the group that was infected the most was adult males. Such a shift in the age distribution of mpox cases (before and after 2022) may have affected the time delays and introduced bias into our comparison of their estimates. Fourth, the differences in epidemiology of mpox infections respective to their clades remain uncertain. Although our estimated mean incubation period for clade I was shorter than the mean for clade II, the difference was not statistically clear and could simply caused by sampling variability (the samples were also relatively small). Overall, the studies aggregated in our meta-analysis were conducted during different time periods and in different geographic locations involving diverse social groups. This variation could introduce variability in public health interventions, diagnostic methods, and reporting practices, potentially affecting estimates of epidemiologic parameters such as the incubation period and serial interval. A cohort-based comparison taking account of observed severity, social status, and other factors could help to address potential biases.

Despite those limitations, our study provides evidence that the incubation period for mpox was similar in 2022 to that of historical outbreaks, whereas the serial interval was shorter. This finding likely reflects both the result of interventions and a shift toward a sexually associated mode of transmission in the 2022 outbreak. Because estimated values of epidemiologic parameters are often used to inform interventions against a range of pathogens, our study highlights the importance of monitoring temporal changes in transmission and disease progression. Effective public health interventions that are tailored to the characteristics of future mpox outbreaks could be crucial for mitigating transmission in the future. Overall, our findings provide useful information to inform evidence-based control strategies to curtail the spread of mpox and other directly transmitted infectious diseases.

Appendix Additional information about incubation period and serial interval of mpox in 2022 global outbreak compared with historical estimates.
