# Supplementary material for: Incubation Period and Serial Interval of Mpox in 2022 Global Outbreak Compared with Historical Estimates
Source: Emerg Infect Dis. 2024 Jun;30(6):1173–81. doi: 10.3201/eid3006.231095 (PMC11138990; doi:10.3201/eid3006.231095)
Supplement: Appendix — Additional information about incubation period and serial interval of mpox in 2022 global outbreak compared with historical estimates. [file 23-1095-Techapp-s1.pdf]

Article DOI: http://doi.org/10.3201/eid3006.231095

*EID cannot ensure accessibility for supplementary materials supplied by authors. Readers who have difficulty accessing supplementary content should contact the authors for assistance.*

# Incubation Period and Serial Interval of Mpox in 2022 Global Outbreak Compared with Historical Estimates

## Appendix

### A. Data Aggregation

a. Inclusion and Exclusion Criteria

Inclusion criteria for selected historical studies required that information on individual pre-2022 mpox cases before were detailed in the text, and that at least one individual case had dates of mpox virus exposure and symptom onset listed, or the period between these two dates listed. Exclusion criteria were: no information on mpox transmission, inaccessible publications, no information on individual mpox cases, and no information on definitive exposure. Inaccessible studies were those that lacked full electronic versions. Studies with overlapping data (i.e., the same individual cases) were included only if they provided additional information about the desired data.

Second, for 2022 and 2023 study inclusion in the meta-analysis, since a standard error value is necessary in addition to the mean incubation period, only peer-reviewed studies with both mean incubation period estimates and their 95% credible interval (CrI) were selected. In addition to the pre-2022 mpox data, we were able to find and use four more datasets. One of these datasets was collected by Viedma-Martinez et al. (*29*), who collected detailed data on mpox cases' dates of exposure and symptom onset that occurred in a tattoo parlor in Cadiz, Spain. However, the authors did not calculate any incubation period estimates, so the estimates were performed in this study. Next was the data used by Miura et al. (*7*). Although Miura et al. had their own incubation period estimates, the statistical methods were re-evaluated and the data were re-analyzed in the present study. Then, the largest available dataset came from Spanish cases collected by Tarin-Vicente et al. (*5*), who did not offer their own Bayesian estimates of the

mean incubation period. Finally, a dataset consisting mostly of U.S. mpox cases, collected by Madewell et al. (*11*), was included in the meta-analysis conducted in this study.

### b. Outcome Measures and Study Selection

The outcome variable within each dataset was the mean estimate of the incubation period. It was measured for all cases within each dataset with information on what day they were exposed to mpox and what day their symptoms began.

For the pre-2022 mpox publications, search results were screened first through titles and abstracts. In this screening stage, publications without full electronic texts available and irrelevant studies were removed. The full texts of the remaining studies were examined and, in the next screening stage, those without the required data—definitive exposure and individual case information—were removed during the screening process. For the global 2022 mpox outbreak studies, new studies were included in the meta-analysis as their data became available. Then, data extraction was conducted for the remaining articles that made it through the screening process. When available, we aimed to collect data about studies' author names, publication date, and for each individual case ID, sex, age, country of origin, disease exposure and symptom onset date, rash onset date, list of symptoms, testing date, disease confirmation date, source of infection, transmission method, and disease status (i.e., confirmed, probable, or suspected).

### c. Data Descriptions and Preparation for Analysis

To meet the inclusion criteria, each case $i$ found in the literature was required to have, at least, partial information on the time window of exposure $(E_{L,i}, E_{R,i})$ and the time window of symptom onset $\left(O_{L,i}, O_{R,i}\right)$. The following adjustments could be made:

- When only the lower boundary of the exposure interval $(E_{L,i})$ was definitive, but the upper boundary $(E_{R,i})$ was unknown or it was later than the upper boundary of the symptom onset interval, we set $E_R$ to the upper boundary of the symptom onset interval $O_{R,i}$, i.e., $E_{R,i} \coloneqq O_{R,i}$ IF $E_{L,i}$ is known AND ($E_{R,i}$ is unknown OR $E_{R,i} > O_{R,i}$);
- All symptom onset dates were definitive, implying the respective time interval is of 1-day long ($O_{R,i} \coloneqq O_{L,i}$).

In total, **42 case records** were aggregated, including **38 records** with completely observed exposure time interval, and **4 records** which were censored.

Similar data preparation techniques were applied to estimate the mean exposure-to-rash incubation period, resulting in **20 case records**, including **4 censored records**.

d. Assessing the Retrieved Datasets

Among all of the currently available mpox incubation period estimates, the present study focused on studies reporting the distribution of incubation periods along with the mean and 95% CrI of that distribution for the hierarchical meta-analyses. Then, the present study created incubation period distributions for datasets where they had not yet been estimated or where estimates could be further improved (e.g., by adjusting for right truncation).

Tarin-Vicente et al. (*5*) Data

Tarin-Vicente et al. (*5*) study aimed to investigate the clinical and virological characteristics of human mpox cases in Spain reported in May–June 2022. They conducted a multicenter, prospective, observational cohort study in three sexual health clinics in Madrid and Barcelona, Spain, and enrolled all consecutive patients with laboratory-confirmed mpox from 11 May through 29 June 2022. The authors collected participant data by conducting interviews using a standard case report form and offered lesion, anal, and oropharynx swabs for RT-PCR testing. In addition to collecting data on date of infection and symptom onset, they also collected data on rash onset dates.

Viedma-Martinez et al. (*38*) Data

Identification of case transmission data of mpox from an outbreak rooted in a tattoo parlor in Cadiz, Spain resulted in 21 confirmed reported cases of mpox, all of whom had visited the tattoo parlor around the same couple of weeks. Most of the cases visited the parlor to get a piercing except for one, who got a tattoo only. To extract and synthesize data for the dates of exposure and dates of symptom onset, we retrieved all available information from the relevant sources.

Miura et al. (7) Data

These data were collected from an outbreak of mpox in the Netherlands and had exact dates of exposure for 13 out of 18 cases, and the exact dates of symptom onset for all cases. Where exposure date was uncertain, a range of dates was available, with a left margin and a right margin.

*Guzzetta et al.* (8) *Data*

An outbreak of confirmed mpox cases in Italy through 8 July 2022 contained complete information (i.e., a known date of exposure and symptom onset) for 15 individual cases. When exposure date was uncertain, a range of earliest exposure and latest exposure was available, for a total of 18 cases included in this study.

**B. Statistical Analysis**

a. Bayesian Framework

Time interval distributions were estimated using Markov chain Monte Carlo (MCMC) sampling techniques for each available dataset. Subsequently, a meta-analysis was conducted by pooling the time interval estimates reported in the literature, along with the estimated values obtained from the present study's analysis, and employing a Bayesian hierarchical/partial pooling model. Bayesian estimation was implemented using Stan software (https://mc-stan.org ). Each run of simulations was consistent of 4 parallel chains with 15,000 posterior draws including 2,500 draws used for tuning-in and disregarded for the final output. Code scripts and all supporting information are publicly available and can be accessed on designated repository: https://github.com/aakhmetz/Mpox-IncubationPeriodSerialInterval-Meta2023

b. Time Interval Estimation

This study involved multiple datasets, each containing information on exposure dates, symptom onset dates, and rash onset dates. Censored data was handled by assigning a weakly informative prior distribution. Then, data lists were constructed for each dataset and descriptively named, including the number of observations ($N$), exposure dates' lower boundaries, $E_L$, symptom or rash onset dates' lower boundaries, $O_L$, and corresponding upper boundaries of these dates, $E_R$ and $O_R$, when available. The same framework was adapted for transmission (infector-infectee) pairs data set, with the only difference being that the exposure dates, $E_L$ and $E_R$, would

stand for the onset dates of the infector, and onset dates, $O_L$ and $O_R$, would stand for the onset dates of the infectee.

For situations where data were missing, the data lists included censored and observed counterparts, such as censored case observations, $N_{cens}$, complete case observations, $N_{obs}$ ($N = N_{obs} + N_{cens}$), censored lower boundary of the exposure, $E_{L,i} = E_{Lcens,i}$, and observed ones, $E_{L,i} = E_{Lobs,i}$, with the defined prior:

$$E_{R,i} - E_{Lcens,i} \sim \text{Exponential}(\text{rate} = 0.1)$$

The time of exposure, $e_i$, and the time of symptom onset, $o_i$, were then assumed to be uniformly distributed within their respective intervals:

$$e_i \sim \text{Uniform}(\text{lower} = E_{L,i}, \text{upper} = E_{R,i} + 1)$$
$$o_i \sim \text{Uniform}(\text{lower} = O_{L,i}, \text{upper} = O_{R,i} + 1)$$

where $i = 1, \ldots, N$.

Each time interval (infection-to-onset and infection-to-rash incubation periods, as well as onset-to-onset and rash-to-rash serial intervals) were fitted to the data using five different models—one of three (gamma, Weibull, or lognormal) distributions, their mixture within a Bayesian mixture model, and the generalized gamma distribution (GGD).

In case of using individual distributions $l$ (gamma, Weibull, lognormal, or GGD; $l = 1, 2, 3, 4$, respectively), the likelihood was a product of probability density functions, $f_l(o - e; \theta)$, at each interval $o_i - e_i$, given by:

$$L_l\big(\theta; D \coloneqq \{E_{\circ,i}, O_{\circ,i}\}\big) = \prod_i \iint_{\Phi_i} \frac{f_l(o_i - e_i; \theta)}{F_l(T + 1 - e_i; \theta)} do_i de_i$$

if the data were right truncated at cut-off date $T$,

$$L_l\big(\theta; D \coloneqq \{E_{\circ,i}, O_{\circ,i}\}\big) = \prod_i \iint_{\Phi_i} f_l(o_i - e_i; \theta) do_i de_i \qquad \textbf{(1)}$$

otherwise. Here, $\circ = \{L, R\}$ and the imposed domain $\Phi_i$ was defined as follows:

$$\Phi_i \coloneqq \big[\{e_i, o_i\}: O_{L,i} \le o_i \le O_{R,i}, E_{L,i} \le e_i \le \big(\{o_i, E_{R,i}\}\big)\big]$$

The $f_l$ was defined by a set of parameters $\theta$, including the mean, $m$, and SD, $s$. All parameters were positive and generic informative priors were assigned to their log-transformed values:

$$\log(m) \sim \text{Normal}(\text{mean} = 2, \text{SD} = 2)$$

$$\log(s) \sim \text{Normal}(\text{mean} = 0, \text{SD} = 2)$$

for all distributions excluding the GGD, and:

$$\log(m) \sim \text{Normal}(\text{mean} = 2, \text{SD} = 2)$$

$$\log(\sigma) \sim \text{Normal}(\text{mean} = 0, \text{SD} = 2)$$

$$\log(a) \sim \text{Normal}(\text{mean} = 0, \text{SD} = 1)$$

otherwise.

In case of the Bayesian mixture model, the overall likelihood, $L$, was given by a mixture of three component likelihoods with respective weights $w_l$:

$$L\big(\theta; D \coloneqq \{E_{\circ,i}, O_{\circ,i}\}\big) = \sum_{l=1,2,3} w_l L_l(\theta; D)$$

These weights were then estimated as part of the fitting process. To facilitate algorithm convergence, two parameters, the mean and standard deviation (SD), were assigned to be common to all three distributions (*49*).

The posterior probability for selecting the distribution $l$ is defined by the expression:

$$\text{Prob}(l) = \frac{w_l L_l(\theta; D)}{L(\theta; D)}$$

In case of analyzing the rash-to-rash serial interval, the cut-off value, $\tau$, was imposed, when all data points with intervals below $\tau$ were truncated. This led to a left-truncated likelihood modified from the form (**1**):

$$L_l\big(\theta; D \coloneqq \{E_{\circ,i}, O_{\circ,i}\}\big) = \prod_i \iint_{\Phi_i} \frac{f_l(o_i - e_i; \theta)}{\tilde{F}_l(\tau - 1; \theta)} do_i de_i$$

where $\tilde{F}(\circ; \theta) = 1 - F(\circ; \theta)$ is a complimentary cumulative distribution function.

**C. Sensitivity Analysis for Rash-To-Rash Serial Interval**

In addition to the eight-day cut-off value considered for **Appendix Figure 1B**, we also calculated the rash-to-rash serial interval under alternative conditions. First, varying the cut-off value between 2 and 10 days yielded a mean rash-to-rash serial interval ranging from 11.9 days to 15.0 days (95% CrI 10.7-16.0 days). Second, we modeled the rash-to-rash interval data using a composition of two distributions: the exponential or scaled standard normal distribution and the serial interval distribution. Both the exponential distribution and the scaled normal distribution as the first component yielded a mean serial interval of 15.1 days (95% CrI 14.2-16.0 days).

**Appendix Table 1.** Consistency in case definition of symptom onset across different studies

| Study | Publication date | Symptom onset definition |
|---|---|---|
| Miura et al. | 2022-06-16 | *Not stated* |
| Tarin-Vicente et al. | 2022-08-08 | Fever, lymphadenopathy, influenza-like symptoms, rash |
| Guzetta et al. | 2022-10-01 | *Not stated,* but mentioning: fever, rash |
| Ward et al. | 2022-10-10 | High temperature, headache, muscle aches, rash |
| Viedma-Martinez et al. | 2023-01-05 | Painful regional inflammatory lymphadenopathy |
| Madewell et al. | 2023-04-23 | Fever, headache, chills, swollen lymph nodes, exhaustion |

**Appendix Table 2.** Infection-to-onset incubation period in studies of mpox before the 2022 outbreak and in the 2022 outbreak*

| Study | # Cases | Publication date | ICC | | | | ICRTC | | | |
|---|---|---|---|---|---|---|---|---|---|---|
| | | | Median, d | Range, d | Mean, d | SD, d | Median, d | Range, d | Mean, d | SD, d |
| Miura et al. | 18 | 2022-06-16 | 8.2 | 2.9–20.3 | 9.1 (7.2–11.6) | 4.6 (3.1–6.4) | 8.4 | 2.8-25.3 | 9.9 (7.3-15.7) | 8.2 (3.2-11.7) |
| Tarin-Vicente et al. | 144 | 2022-08-08 | 7.5 | 2.3–15.6 | 7.8 (7.3–8.4) | 3.5 (3.0–3.9) | 7.6 | 2.2-15.7 | 7.9 (7.3-8.5) | 3.5 (3.1-4.0) |
| Guzetta et al. | 30 | 2022-10-01 | 7.0 | 1.0–23.7 | 8.4 (6.4–11.1) | 6.4 (3.7–9.1) | 7.1 | 0.9-27.8 | 9.0 (6.5-13.8) | 9.4 (3.7-14.4) |
| Ward et al. | 54 | 2022-10-10 | 6.5 | 0.7–20.5 | 7.6 (6.5–8.9) | 5.4 (4.4–6.7) | 6.6 | 0.7-21.0 | 7.7 (6.6-9.2) | 5.6 (4.4-7.1) |
| Viedma-Martinez et al. | 21 | 2023-01-05 | 7.6 | 3.4–16.0 | 8.1 (6.8–9.6) | 3.3 (2.7–4.5) | Not required | | | |
| Madewell et al. | 36 | 2023-04-23 | n/a | n/a | 5.6 (4.3–7.8) | 4.4 (2.8–8.7) | Insufficient information | | | |
| Ogoina et al. | 12 | 2023-05-17 | 5.1 | 0.8–20.8 | 6.6 (4.2–10.8) | 6.7 (2.3–12.0) | Not required | | | |
| McFarland et al. | 122 | 2023-07-06 | 8.7 | 1.7–23.9 | 9.8 (8.7–10.9) | 5.9 (4.8–6.9) | Not required | | | |
| Zhang et al. | 75 | 2023-09-11 | 6.9 | 4.1–20.5 | 8.2 (6.9–9.4) | 5.5 (n/a–n/a) | 6.9 | 4.1-20.2 | 8.1 (6.9-9.3) | 5.5 (n/a-n/a) |
| Alvarez et al. | 11 | 2023-12-07 | 8.6 | 1.5–24.2 | 9.7 (6.6–14.7) | 7.0 (2.9–11.8) | Insufficient information | | | |
| Historical data | 42 | Pre-2022 | 7.4 | 1.3–19.8 | 8.2 (6.7–10.0) | 4.6 (3.4–6.5) | Not required | | | |
| Clade I | 16 | Pre-2022 | 6.5 | 0.8–17.9 | 7.3 (5.0–10.2) | 5.6 (2.6–8.3) | Not required | | | |
| Clade II | 22 | Pre-2022 | 7.7 | 1.4–23.5 | 8.9 (6.6–11.7) | 5.1 (3.5–8.4) | Not required | | | |

*The studies are ordered by their publication dates. Mean and SD are represented by their posterior means and 95% CrI; median and range are represented only by their posterior means. ICC, interval censoring corrected model; ICRTC, interval censoring and right truncation corrected model; Range, 95% CrI as the interval between 2.5th and 97.5th percentiles of the posterior distribution; n/a, not available as it was not stated in the original study.

**Appendix Table 3.** Infection-to-rash incubation period in studies of mpox before the 2022 outbreak and in the 2022 outbreak*

| Study | # Cases | Publication date | ICC | | | | ICRTC | | | |
|---|---|---|---|---|---|---|---|---|---|---|
| | | | Median, d | Range, d | Mean, d | SD, d | Median, d | Range, d | Mean, d | SD, d |
| Tarin-Vicente et al. | 143 | 2022-08-08 | 8.7 | 2.3–17.7 | 9.0 (8.4–9.7) | 4.0 (3.5–4.5) | 8.8 | 2.3–18.1 | 9.2 (8.5–9.9) | 4.1 (3.6–4.8) |
| Viedma-Martinez et al. | 19 | 2023-01-05 | 9.8 | 3.7–16.0 | 9.8 (8.5–11.2) | 3.1 (2.2–4.6) | Not required | | | |
| Madewell et al. | 35 | 2023-04-23 | n/a | n/a | 7.5 (6.0–9.8) | 4.9 (3.2–8.8) | Insufficient information | | | |
| Historical data | 28 | Pre-2022 | 9.7 | 3.4–20.5 | 10.3 (8.5–12.3) | 4.3 (3.1–5.9) | Not required | | | |
| Clade I | 16 | Pre-2022 | 8.9 | 2.5–19.4 | 9.4 (7.2–12.1) | 4.1 (2.6–6.6) | Not required | | | |
| Clade II | 12 | Pre-2022 | 11.1 | 3.8–23.6 | 11.7 (8.8–15.2) | 5.0 (3.1–8.1) | | | | |

*The studies are ordered by their publication dates. Mean and SD are represented by their posterior means and 95% CrI; median and range are represented only by their posterior means. ICC, interval censoring corrected model; ICRTC, interval censoring and right truncation corrected model; Range, 95% CrI as the interval between 2.5th and 97.5th percentiles of the posterior distribution; n/a, not available as it was not stated in the original study.

**Appendix Table 4.** Serial interval inferred from onset-to-onset case interval data in studies of mpox before the 2022 outbreak and in the 2022 outbreak*

| | | | ICC | | | | ICRTC | | | |
|---|---|---|---|---|---|---|---|---|---|---|
| Study | # of pairs | Publication date | Median, d | Range, d | Mean, d | SD, d | Median, d | Range, d | Mean, d | SD, d |
| Guzetta et al. | 18 | 2022-10-01 | 6.5 | 0.3-29.3 | 8.2 (5.5-13.8) | 6.3 (4.2-18.0) | 6.8 | 0.3-47.8 | 8.9 (5.7-40.7) | 7.2 (4.3-138.5) |
| Guo et al. | 30 | 2022-10-22 | n/a | n/a | 4.3 (1.9-7.0) | 2.6 (1.1-3.2) | 5.5 | n/a | 5.6 (1.7-10.4) | 1.5 (0.4-2.4) |
| Ward et al. | 54 | 2022-10-10 | 5.0 | 0.1-32.2 | 8.0 (6.5-9.9) | 9.0 (7.0-11.7) | 5.7 | 0.1-38.2 | 9.5 (7.4-12.3) | 10.9 (8.0-15.0) |
| Miura et al. | 21 | 2023-01-05 | n/a | n/a | 10.1 (6.6-14.7) | 6.1 (4.6-8.0) | | not required | | |
| Madewell et al. | 36 | 2023-04-23 | n/a | n/a | 8.5 (7.3-9.9) | 5.0 (4.0-6.4) | | no difference | | |
| Zhang et al. | 121 | 2023-09-11 | 8.8 | 5.2-26.9 | 10.5 (8.9-12.1) | 9.0 (nan-nan) | 8.8 | 5.2-25.8 | 10.4 (8.8-12.0) | 8.9 (nan-nan) |
| Historical data | 42 | Pre-2022 | 13.5 | 6.9-25.5 | 14.2 (12.5-16.2) | 5.7 (4.1-6.9) | | not required | | |

*The studies are ordered by their publication dates. Mean and SD are represented by their posterior means and 95% CrI; median and range are represented only by their posterior means. ICC, interval censoring corrected model; ICRTC, interval censoring and right truncation corrected model; Range, 95% CrI as the interval between 2.5th and 97.5th percentiles of the posterior distribution; n/a, not available as it was not stated in the original study.

**Appendix Table 5.** Serial interval inferred from rash-to-rash case interval data in studies of mpox before the 2022 outbreak and in the 2022 outbreak*

| | | | ICC | | | | ICRTC | | | |
|---|---|---|---|---|---|---|---|---|---|---|
| Study | # of pairs | Publication date | Median, d | Range, d | Mean, d | SD, d | Median, d | Range, d | Mean, d | SD, d |
| Madewell et al. | 40 | 2023-04-23 | n/a | n/a | 7.0 (5.8-8.4) | 4.2 (3.2-5.6) | | no difference | | |
| Historical data | 28 | Pre-2022 | 14.5 | 6.1-21.7 | 14.3 (13.2-15.3) | 4.0 (3.4-4.8) | | *not required* | | |

*The studies are ordered by their publication dates. Mean and SD are represented by their posterior means and 95% CrI; median and range are represented only by their posterior means. ICC, interval censoring corrected model; ICRTC, interval censoring and right truncation corrected model; Range, 95% CrI as the interval between 2.5th and 97.5th percentiles of the posterior distribution; n/a, not available as it was not stated in the original study.

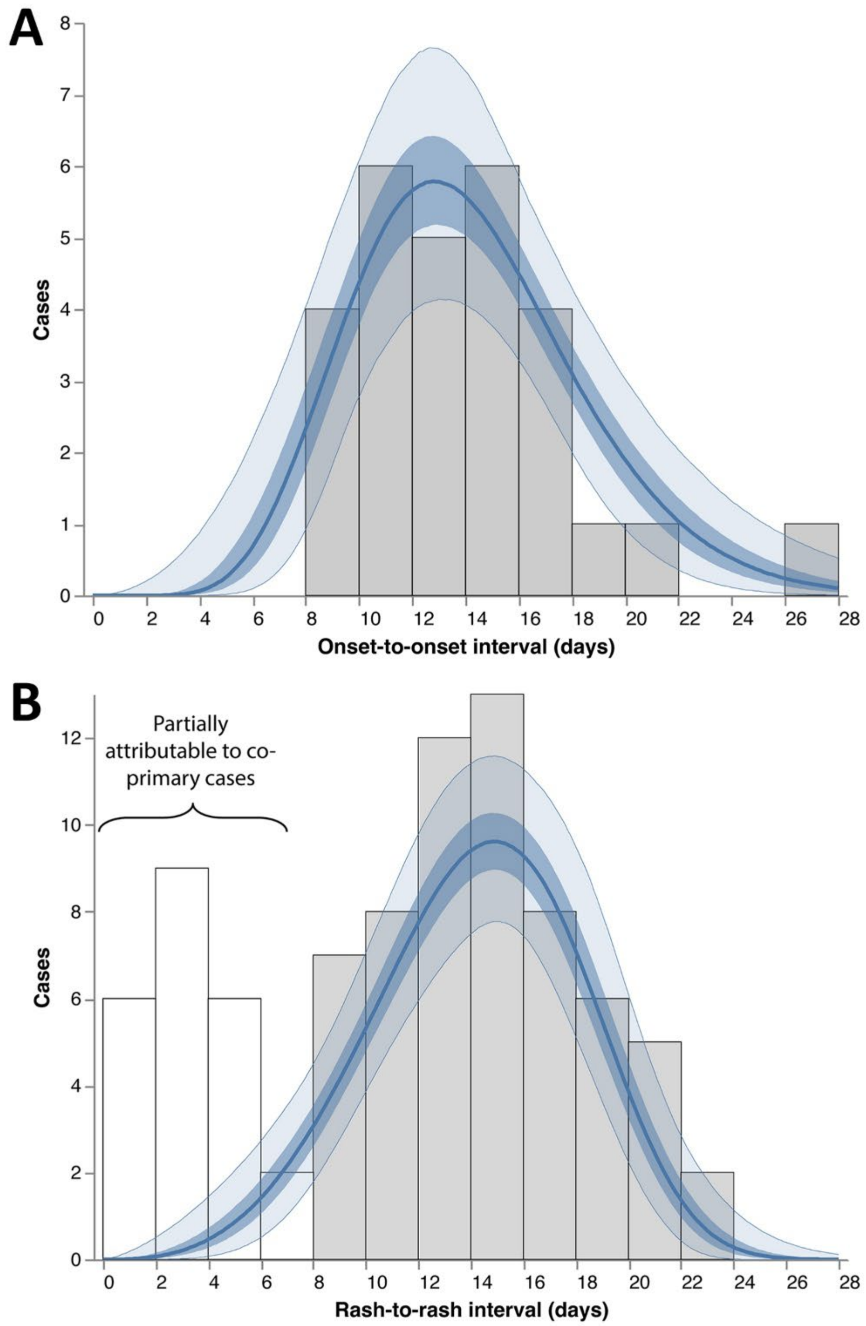


**Appendix Figure 1.** Fitting the serial interval distribution based on symptom onset (A) and rash onset (B) for historical (pre-2022) data. The serial interval is depicted in blue (the solid line represents the median and darker shaded area represents the interquartile range, while the lighter shaded area represents the 95% credible interval). In (A), the bins indicate the onset-to-onset intervals attributable to linked transmission (infector-infectee) pairs. In (B), the filled bins indicate the rash-to-rash intervals likely attributable to transmission pairs, while unfilled bins indicate the rash-to-rash intervals attributable either to co-primary cases or, more likely, to two independent sources of infection. In this situation, a left truncated likelihood was employed for the analysis.

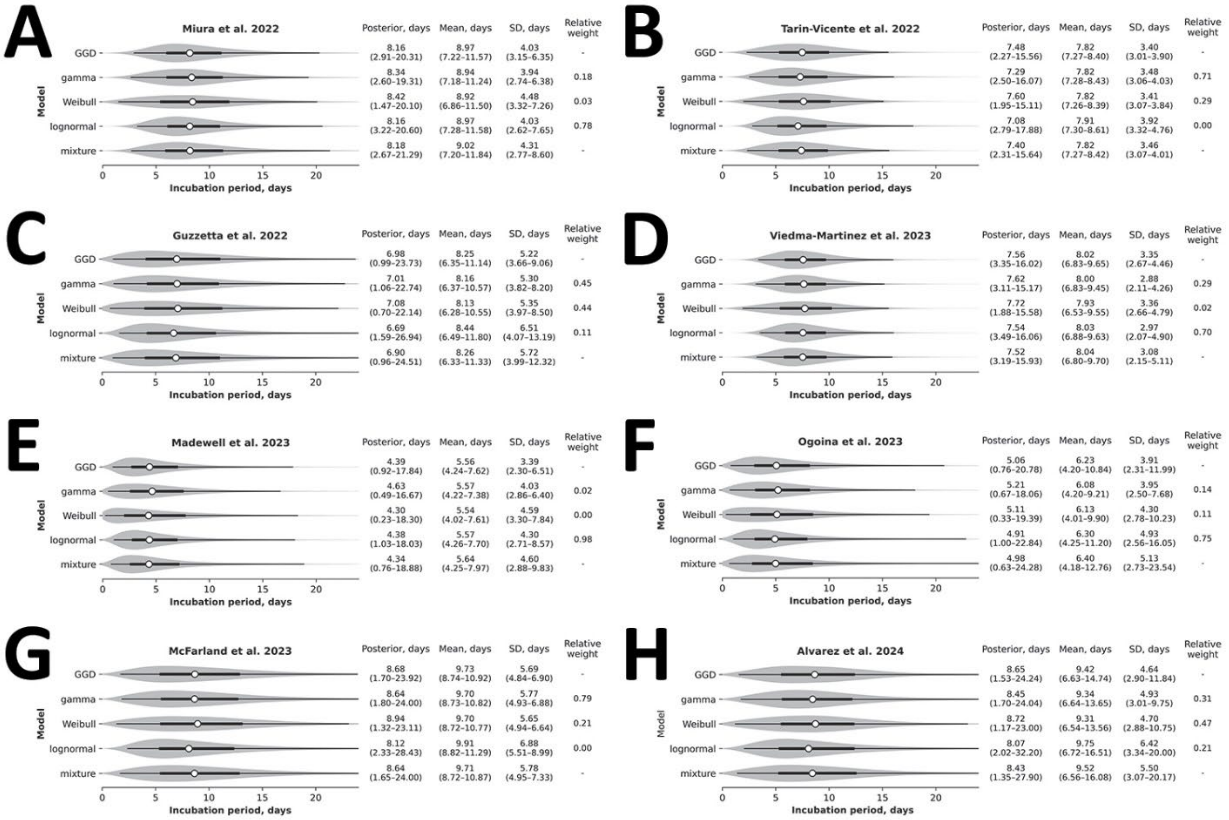


**Appendix Figure 2.** Estimated infection-to-onset incubation periods fitted using the generalized gamma distribution (GGD), gamma distribution, Weibull distribution, lognormal distribution, or the mixture of the last three distributions (gamma, Weibull, and lognormal) without correcting for right truncation and for various studies. The derived posterior predictive incubation period, mean incubation period and its standard deviation are shown in three columns by their posterior medians and 95% CrI. The last column shows the relative weight of each distribution among three, determined within the Bayesian mixture model. The sum of posterior medians was normalized to one.
